# The cystic fibrosis transmembrane conductance regulator is an extracellular chloride sensor

**DOI:** 10.1007/s00424-014-1618-8

**Published:** 2014-10-04

**Authors:** Steven D. Broadbent, Mohabir Ramjeesingh, Christine E. Bear, Barry E. Argent, Paul Linsdell, Michael A. Gray

**Affiliations:** 1 Epithelial Research Group, Institute for Cell & Molecular Biosciences, Newcastle University, Newcastle upon Tyne, NE2 4HH UK; 2 Hospital for Sick Children and Departments of Biochemistry and Physiology, University of Toronto, Toronto, ON Canada; 3 Department of Physiology & Biophysics, Dalhousie University, Halifax, NS Canada

**Keywords:** CFTR, Channel gating, Epithelial ion transport, Electrophysiology

## Abstract

The cystic fibrosis transmembrane conductance regulator (CFTR) is a Cl^−^ channel that governs the quantity and composition of epithelial secretions. CFTR function is normally tightly controlled as dysregulation can lead to life-threatening diseases such as secretory diarrhoea and cystic fibrosis. CFTR activity is regulated by phosphorylation of its cytosolic regulatory (R) domain, and ATP binding and hydrolysis at two nucleotide-binding domains (NBDs). Here, we report that CFTR activity is also controlled by extracellular Cl^−^ concentration ([Cl^−^]_o_). Patch clamp current recordings show that a rise in [Cl^−^]_o_ stimulates CFTR channel activity, an effect conferred by a single arginine residue, R899, in extracellular loop 4 of the protein. Using NBD mutants and ATP dose response studies in WT channels, we determined that [Cl^−^]_o_ sensing was linked to changes in ATP binding energy at NBD1, which likely impacts NBD dimer stability. Biochemical measurements showed that increasing [Cl^−^]_o_ decreased the intrinsic ATPase activity of CFTR mainly through a reduction in maximal ATP turnover. Our studies indicate that sensing [Cl^−^]_o_ is a novel mechanism for regulating CFTR activity and suggest that the luminal ionic environment is an important physiological arbiter of CFTR function, which has significant implications for salt and fluid homeostasis in epithelial tissues.

## Introduction

**Fig. 1:**
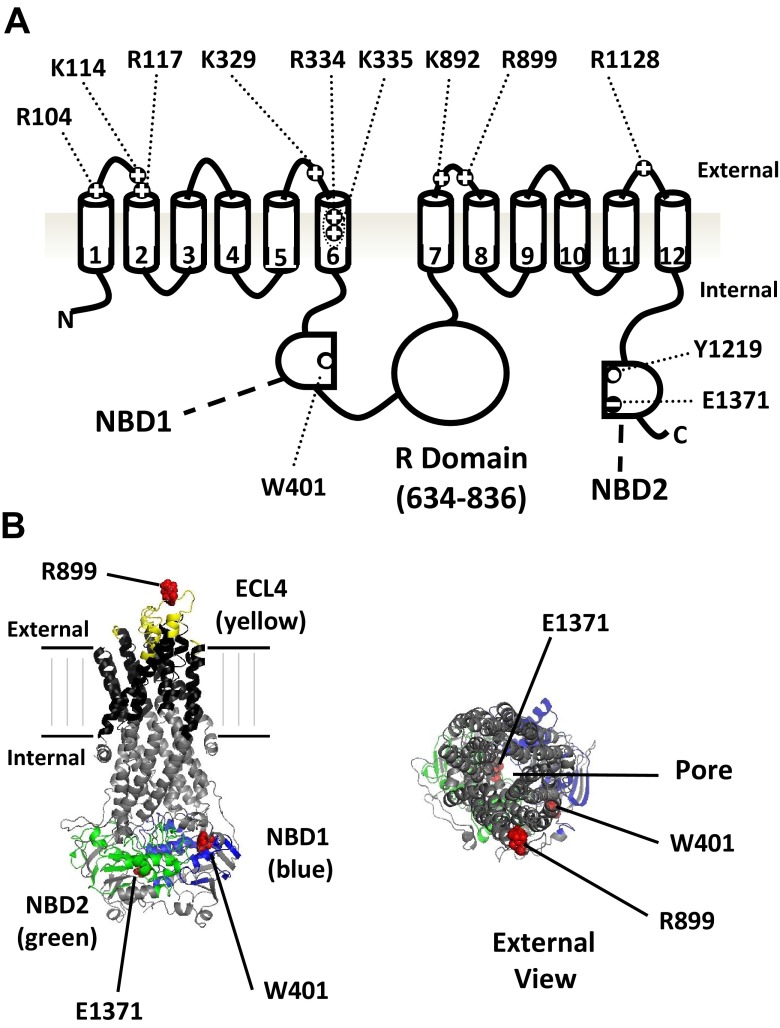
Molecular structure of CFTR. **a** Cartoon of the CFTR protein showing the location and charge of the all amino acids mutated in this study. Note that the extracellular loops are not drawn to scale. **b** Three-dimensional homology model of CFTR based on the work of Dalton et al. [14], using PyMol software indicating the relative positions of R899, W401 and E1371 residues to other regions of note. Note that the homology model lacks the R domain and that in the right hand diagram, the extracellular surface of CFTR is orientated to face the reader.

The cystic fibrosis transmembrane conductance regulator (CFTR) is an anion channel which plays a crucial role in regulating salt and fluid transport in the airways, sweat glands and the gastrointestinal and genital tracts [36, 26]. Loss of function mutations in CFTR cause the fatal inherited disease cystic fibrosis (CF) [32], while overactive CFTR in the GI tract leads to secretory diarrhoea [41]. CFTR is composed of two transmembrane domains (TMDs), each consisting of six transmembrane spans connected by extracellular loops (ECLs), plus two cytosolic nucleotide-binding domains (NBDs) and a large regulatory (R) domain [36, 32]. There are also a number of positively charged residues in the ECLs and outer pore [32, 19]. The positions of these residues and the other major domains of CFTR are shown in Fig. 1a. Opening of CFTR requires cAMP/PKA-dependent phosphorylation of the R domain followed by the binding of two ATP molecules; one to site 1 of NBD1 and one to site 2 of NBD2, which induces dimerisation of the NBDs in a head-to-tail fashion and leads to opening of the ‘gate’ located in the TMDs (see [23] for a recent review). Channel closing is caused by ATP hydrolysis at site 2 and the subsequent release of ADP and Pi drives disassembly of the NBD dimer [16, 40]. Because site 1 lacks a number of essential (conserved) residues necessary for efficient nucleotide hydrolysis, ATP remains bound at this site much longer than at site 2. Therefore, it is believed that events at site 2 mainly terminate channel openings [6, 2, 5]. However, recent work indicates that ATP binding to site 1 also contributes to the stability of the open state [49], and therefore has an important, but less well-defined, role in CFTR gating.

Extracellular (i.e. luminal in the physiological situation) Cl^−^ concentration ([Cl^−^]_o_) can regulate the function of CFTR. We have shown that an increase in [Cl^−^]_o_ enhances CFTR activity, both in cell-free, inside out, as well as whole cell patch clamp recordings [29, 44], indicating that a change in channel gating, not channel numbers, underlies the response. Others have reported that a reduction in [Cl^−^]_o_ alters the HCO_3_^−^/Cl^−^ permeability ratio and the conductance of the channel [35, 27]. Because most CFTR-expressing epithelia secrete substantial amounts of Cl^−^ and HCO_3_^−^ (up to 140 mM HCO_3_^−^ in the case of the pancreas [3]), and because the concentrations of these anions in the luminal fluid varies under physiological conditions [39, 25, 33, 37, 43], such studies have led to the speculation that CFTR may be regulated by the Cl^−^ content of its own secretions [44, 27]. However, how [Cl^−^]_o_ is sensed by epithelial cells and how changes in [Cl^−^]_o_ are communicated to CFTR in order to modulate its gating, permeability and conductance are fundamentally important questions that have not been answered.

The goal of this study was to establish if CFTR itself could act as an extracellular Cl^−^ sensor and control its own gating. To address this question, wild type (WT) and mutant forms of CFTR were heterologously expressed in mammalian cells and the response to changes in [Cl^−^]_o_ studied using the patch clamp technique. CFTR was also purified and the effect of [Cl^−^] on its ATPase activity measured to determine if Cl^−^ sensing constitutes an intrinsic property of the CFTR protein.

## Materials and methods

### Cell culture, plasmids and transfection

Plasmids were constructed using either the pIRES2-EGFP (CLONTECH Laboratories, Inc., Mountain View, CA, USA) or pBudCE4.1 (Invitrogen, Burlington, ON, Canada) as described previously [18, 7]. Human embryonic kidney (HEK 293) cells were grown in high-glucose Dulbecco’s modified Eagle’s medium (DMEM) with 10 % fetal calf serum (FCS), and 1 % penicillin and streptomycin in a 5 % CO_2_ humidified atmosphere at 37 °C. HEK cells grown on glass coverslips were transiently transfected with WT or mutant forms of human CFTR. Briefly, plasmid DNA was pre-complexed with OptiMEM-with-GlutaMAX (Invitrogen, Paisley, UK) and Lipofectamine 2000 (Invitrogen, Paisley, UK) for 15 min. Complexed DNA was then diluted in supplement-free medium to ∼0.5 μg/ml and added to the cells. After 6 h at 37 °C and 5 % CO_2_, the medium was replaced with OptiMEM containing 5 % FCS. Transfected cells were identified by epifluorescence and used 2 to 4 days after transfection. Expression levels of the DeltaR-CFTR constructs were augmented by incubating cells at 27 °C in 5 mM sodium butyrate after transfection [22]. For patch clamp experiments, cells were studied at ∼40–50 % confluency on glass coverslips.

### Electrophysiology

Fast whole cell patch clamp recordings (fWCR) and data analysis were performed as described previously [44]. Whole cell currents were recorded at room temperature with an Axopatch 200B amplifier (Molecular Devices Inc., Wokingham, UK) and steady-state current-voltage (I-V) relationships obtained by holding the membrane potential (*V*_m_) at 0 mV and clamping to ±100 mV in 20 mV increments for 250 ms, with a 1-s interval between each pulse. Current amplitudes were calculated at the reversal potential (*E*_rev_) to ±60 mV and normalised to cell capacitance (pF). The *E*_rev_ was obtained by interpolation from I-V plots (*x*-axis intercept obtained by fitting a fourth-order polynomial to the I-V data points). After establishing a fWCR, the standard experimental protocol was to measure: (1) control currents in high (155.5 mM) Cl^−^ bath solution, followed by forskolin (10 μM)-stimulated currents (measured after at least 5-min exposure to forskolin, FSK) in (2) high (155.5) mM Cl^−^, (3) low (35.5 mM) Cl^−^ and (4) high (155.5) mM Cl^−^. The percentage stimulation of CFTR activity by Cl^−^ was calculated by comparing the current amplitude at −60 mV from the *E*_rev_, in low Cl^−^, with the average of the current amplitudes in high Cl^−^ immediately before and after exposure to low Cl^−^. We specifically chose to report inward currents because this represents the most relevant physiological situation (i.e. Cl^−^ efflux). Using this approach, the calculated percentage stimulation of CFTR activity by external Cl^−^ is independent of any change in driving forces under the low and high Cl^−^ conditions [44]. The pipette (intracellular) solution contained (in mM) 139 CsCl, 2 MgCl_2_, 5 ethylene glycol-bis(B-aminoethylether)-N,N,N′,N′-tetraacetic acid (EGTA), 10 HEPES, 5 glucose, 1 Na_2_ATP and 0.1 Na_2_GTP (pH = 7.2) with CsOH. In the experiments using different ATP concentrations or ATP analogues, the MgCl_2_ concentration was altered to maintain a 1 mM free Mg^2+^ concentration calculated on a 1:1 basis, with CsCl concentration adjusted to maintain Cl^−^ concentration and osmolarity. The high-chloride (155.5 mM) bath (extracellular) solution contained (in mM) 145 NaCl, 4.5 KCl, 1 MgCl_2_, 2 CaCl_2_, 10 HEPES and 15 l-glutamic acid monosodium salt hydrate (pH = 7.4) with NaOH. For the 35.5-mM chloride solution, 120 mM NaCl was replaced with 120 Na aspartate. Forskolin was purchased from Tocris Bioscience (Bristol, UK) and all other chemicals from Sigma-Aldrich (Poole, UK).

### Purification and reconstitution of CFTR

The method for purification of CFTR from transfected Sf9 cells using perfluoro-octanoate (PFO) has been described previously [15]. The following modification was included. At the point of protein elution from nitrilotriacetic acid beads during fast protein liquid chromatography, the program was interrupted and the protein was subjected to detergent exchange. The column was first washed with 10 ml of a buffer containing 25 mM phosphate, 50 mM NaCl, 2 % PFO at pH 8.0. A second wash consisted of 50 ml of buffer containing 25 mM HEPES, 100 mM NaCl, 1 mM *n*-dodecyl-β-d-maltopyranoside (DDM) at pH 7.4. The final elution buffer (25 ml) contained 600 mM imidazole. The eluted protein was washed with imidazole-free buffer and concentrated using an Amicon Ultra centrifugal filter device (Milipore Corp. Billerica, MA, USA), (100 kDa cutoff) to bring the imidazole concentration below 1 mM and the protein concentration not greater than 0.2 mg/ml. The protein was phosphorylated at 4 °C overnight in the presence of 2 mM ATP, 5 mM Mg^2+^ and 200 nM of protein kinase A catalytic subunit (PKA; Promega Corp. Madison, WI, USA). CFTR was reconstituted into 5 mg of phosphatidyl-ethanolamine (PE):phosphatidylserine (PS):phosphatidylcholine (PC):ergosterol, 5:2:1:1 (*w*/*v*) or 5 mg Egg PC by incubation in the presence of DDM and lipid for 30 min, followed by passage through an Extracti-Gel D detergent-binding column (Pierce Corp. Rockford, IL, USA). Purified CFTR was quantitated using an ELISA assay described below and mixed at a ratio of ∼1:1,200 (protein:lipid, *w*/*w*) with Egg PC and DDM.

### ATPase assay of purified CFTR

The ATPase activity of purified and PKA phosphorylated WT CFTR in DDM micelles was measured as the production of inorganic phosphate in the presence of Na-gluconate/NaCl (100 mM/50 mM NaCl) or NaCl alone (150 mM) with approximately 2 μg CFTR protein aliquot per reaction. Three independent purifications of WT CFTR were studied. Phosphate release was determined using the PiPer fluorescent assay (Invitrogen, Burlington, ON, Canada), 1 h after initiation of the ATPase activity assay by the addition of 0.05–1.0 mM ATP at 37 °C, in the presence of either Na-gluconate/NaCl (100 mM/50 mM) or NaCl (150 mM), using approximately 2 μg CFTR protein aliquot per reaction. This assay enables the rapid evaluation of multiple experimental conditions simultaneously. A caveat of studies of membrane proteins in detergent micelles is the possibility that the detergent environment does not completely recapitulate the interactions conferred by the lipid bilayer on the membrane domains. However, DDM is commonly employed in crystallography studies of the structure of membrane proteins and, more specifically, we found that the ATPase activity of DDM-solubilised CFTR was regulated by PKA-dependent phosphorylation as expected from our previous studies of lipid reconstituted CFTR [31].

### Modelling of CFTR

A three-dimensional homology model of CFTR was created indicating the relative positions of R899, W401 and E1371 residues (Fig. 1b), based on the work of Dalton et al. [14], using PyMol software (The PyMOL Molecular Graphics System, Version 1.1 eval Schrödinger, LLC)

### Statistics

Data are presented as mean ± SEM. Significance of difference between mean values of different constructs was tested either by one-way ANOVA with Bonferroni’s Multiple Comparison Test or Kruskal-Wallis followed with Dunn’s Multiple Comparison test if Anderson-Darling normality testing of the data showed the values were not normally distributed.

## Results

### Role of ECL residues in the gating of CFTR by extracellular Cl^−^ concentration

**Fig. 2:**
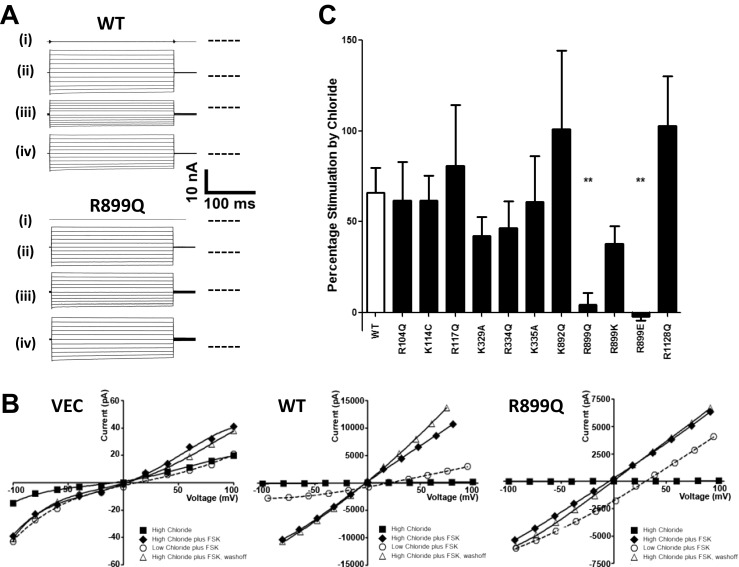
Arginine residue 889 in extracellular loop 4 of CFTR is essential for [Cl^−^]_o_ sensing. **a** Representative fWCR current recordings measured between ±100 mV in 20 mV steps from HEK cells transfected with wild type (*WT*) CFTR and R899Q CFTR, as indicated. The current traces are from the top down: (*i*) unstimulated in 155.5 mM [Cl^−^]_o_, (*ii*) forskolin (FSK)-stimulated in 155.5 mM [Cl^−^]_o_, (*iii*) FSK-stimulated in 35.5 mM [Cl^−^]_o_ and (*iv*) FSK-stimulated in 155.5 mM [Cl^−^]_o_. *Dotted line* to the right of the current traces indicates zero current level. **b** Representative I-V plots for the Vector Control (*VEC*), WT and R899Q CFTR, for unstimulated in 155.5 mM [Cl^−^]_o_ (*black squares*); FSK-stimulated in 155.5 mM [Cl^−^]_o_ (*black rhombus*); FSK-stimulated in 35.5 mM [Cl^−^]_o_ (*white circles*) and FSK-stimulated in 155.5 mM [Cl^−^]_o_ washoff (*white triangles*). I-V plots were obtained from whole cell currents in **a**. All constructs, except the vector control, showed positive *E*_rev_ shifts (see Table 1) on switching to low [Cl^−^]_o_ consistent with the expression of a Cl^−^-selective conductance. Note the different *y*-axis scales for the respective I-Vs. **c** Percentage stimulation of FSK-activated currents by [Cl^−^]_o_ for WT CFTR (*n* = 24) and for different extracellular loop mutants (see Fig. 1a) (*n* = 4–9). Percent stimulation was calculated from I-V data at −60 mV from the reversal potential, as described in “Materials and methods.” Data are means ± SEM. **p < 0.01 compared to WT CFTR

To establish if CFTR was itself an extracellular Cl^−^ sensor, the positively charged residues in the ECLs of CFTR (R104, K114, R117, K329, K892, R899, R1128) (Fig. 1a), as well as R334 and K335 located at the putative outer mouth of the channel pore [19] (Fig. 1a), were individually mutated to neutral amino acids and the mutant channels tested for their ability to respond to changes in [Cl^−^]_o_. Note that our previous work found that gating of CFTR by [Cl^−^]_o_ was voltage-independent [44], and therefore unlikely to involve residues further within the channel pore that experience the transmembrane electric field. Figure 2a illustrates the response of cAMP-activated WT CFTR (cAMP levels increased by FSK) to an increase in [Cl^−^]_o_ from 35.5 to 155.5 mM, which stimulated whole cell currents by 66.1 ± 13.7 % (*n* = 24), as we have previously reported [44]. Figure 2b, c shows that removing the positive charge at position 899 (R899Q) completely abolished [Cl^−^]_o_ sensing by CFTR (high Cl^−^ stimulation; 4.3 ± 6.6 %, *n* = 7), whereas all the other ECL charge neutralising mutants had no significant effect on the response (Fig. 2c). Furthermore, introducing a lysine residue in place of arginine (R899K) maintained the response to [Cl^−^]_o_ (Fig. 2c) while mutating R899 to the negatively charged glutamic acid (R899E) did not, clearly showing that the positive charge at position 899 in ECL4 of CFTR is essential for [Cl^−^]_o_ sensing. Typical I-V plots obtained in this series of experiments for WT, R899Q and the vector control are shown in Fig. 2b. The effect of FSK on the CFTR currents and the *E*_rev_ shifts on switching to low [Cl^−^]_o_ (an indication of the Cl^−^ selectivity of the FSK-activated currents) for a number of different CFTR constructs and experimental conditions are summarised in Table 1.

**Table 1:** Summary of the FSK stimulation of whole cell currents and *E*_rev_ shifts observed with the CFTR constructs used in this study

CFTR Construct	*n*	FSK Stimulation (% ± SEM)	Erev shift (mV ± SEM)
WT (50 μM ATP)	5	180 ± 96	15.0 ± 3.6
WT (100 μM ATP)	6	12,000 ± 6,000	15.2 ± 3.0
WT (300 μM ATP)	8	1,200 ± 600	17.0 ± 3.0
WT (1 mM ATP)	24	13,000 ± 6,000	23.7 ± 1.8
WT (1.3 mM ATP)	9	1,400 ± 900	16.7 ± 2.6
WT (2 mM ATP)	24	6,100 ± 5,300	16.7 ± 1.6
WT (5 mM ATP)	7	1,600 ± 1,000	20.1 ± 4.4
WT (50 μM ATP + 50 μM P-ATP)	7	224 ± 130	15.3 ± 1.0
WT + Genistein	4	7,600 ± 5,200	26.1 ± 5.4
WT + AMP-PNP	5	2,800 ± 2,500	21.8 ± 5.5
WT (3 mM MgCl_2_)	7	28,000 ± 17,000	18.3 ± 3.1
R104Q	5	4,600 ± 1,600	28.6 ± 4.7
K114C	5	12,000 ± 6,700	29.2 ± 3.0
R117Q	4	33,000 ± 20,000	30.1 ± 3.4
K329A	5	13,000 ± 10,000	33.7 ± 2.1
R334Q	9	13,000 ± 6,700	27.3 ± 2.9
K335A	5	3,200 ± 1,500	20.8 ± 7.1
W401G	7	2,600 ± 1,800	18.5 ± 4.8
Delta-R (No Stim)	5	-	25.1 ± 2.7
Delta-R (No FSK, Genistein)	5	140 ± 13	22.7 ± 3.0
Delta-R (FSK, No Genistein)	4	89 ± 14	15.6 ± 6.0
Delta-R (FSK + Genistein)	6	639 ± 432	25.1 ± 4.9
Delta-R-E1371S (No FSK)	9	-	21.4 ± 4.8
Delta-R-E1371S (FSK)	4	2,600 ± 1,400	15.3 ± 4.7
K892Q	7	16,000 ± 9,500	36.8 ± 4.8
R899E	4	1,200 ± 400	25.0 ± 2.7
R899K	4	1,600 ± 900	26.6 ± 2.9
R899Q	7	5,400 ± 2,800	30.0 ± 1.3
R899Q + AMP-PNP	4	72,000 ± 50,000	15.2 ± 2.8
R899Q-E1371Q (No FSK)	4	-	18.4 ± 5.9
R899Q-E1371Q (FSK)	6	107 ± 48	15.6 ± 3.0
R1128Q	6	14,000 ± 6,100	41.1 ± 4.2
Y1219G	6	3,200 ± 2,500	19.2 ± 3.3
E1371Q (No FSK)	6	-	25.5 ± 3.5
E1371Q (FSK)	8	−28 ± 9	22.3 ± 4.0
E1371Q (FSK, No ATP, No GTP)	8	270 ± 130	19.4 ± 4.5
E1371Q + AMP-PNP (No FSK)	4	-	24.7 ± 6.5
E1371Q + AMP-PNP (FSK)	8	180 ± 170	17.4 ± 4.0
Vector Control	4	15 ± 38	-

FSK stimulation was calculated as the percentage increase in current density at −60 mV from the *E*_rev_, after 5-min exposure to 10 μM FSK. The *E*_rev_ shift is the change in *E*_rev_ value on switching from 155.5 to 35.5 mM [Cl^−^]_o_, in the presence of forskolin (see “Material and methods”). The *E*_rev_ shift predicted by the Nernst equation for a perfectly selective Cl^−^ conductance under these conditions is 37.5 mV. Note that because the vector control currents were very small (Fig. 2b), the *E*_rev_ shift is unreliable and not included in the Table

### Role of ATP hydrolysis and phosphorylation in [Cl^−^]_o_ gating of CFTR

**Fig. 3:**
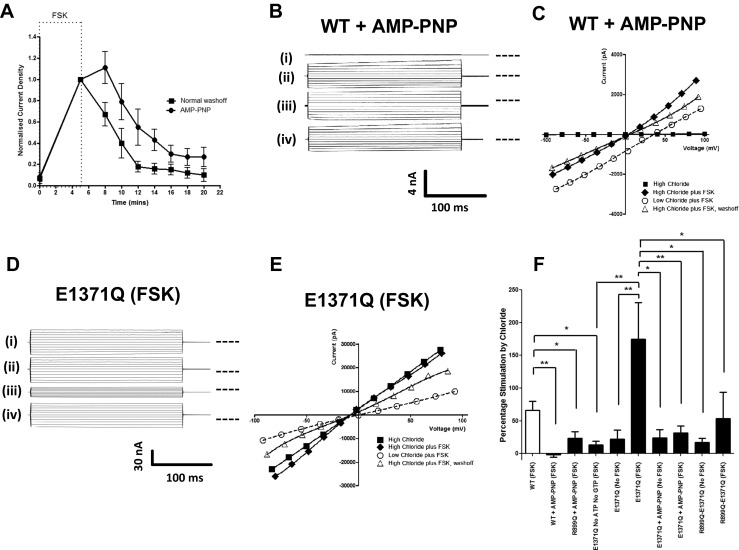
Gating of CFTR by [Cl^−^]_o_ requires ATP hydrolysis and phosphorylation. **a** AMP-PNP locks WT CFTR in the open state. Normalised whole cell current density is plotted against time during FSK stimulation (indicated by the *dashed line*) and the subsequent washoff in the absence (*squares*, *n* = 4) and presence (*circles*, *n* = 4) of 1 mM AMP-PNP in the pipette solution. **b, d** Representative fWCR current recordings measured between ±100 mV in 20 mV steps from HEK cells transfected with WT CFTR plus AMP-PNP and E1371Q CFTR, as indicated. The current traces are from the top down: (*i*) unstimulated in 155.5 mM [Cl^−^]_o_, (*ii*) forskolin (FSK)-stimulated in 155.5 mM [Cl^−^]_o_, (*iii*) FSK-stimulated in 35.5 mM [Cl^−^]_o_ and (*iv*) FSK-stimulated in 155.5 mM [Cl^−^]_o_. *Dotted line* to the right of the current traces indicates zero current level. **c, e** Representative I-V plots for the data presented in **b** and **d. f** Percentage stimulation of either basal or FSK-activated currents by [Cl^−^]_o_ for WT CFTR (*n* = 24), the ECL4 mutant R899Q, the hydrolysis-deficient mutant E1371Q and the double-mutant R899Q-E1371Q (see Fig. 1) under different conditions as indicated (*n* = 4–8). Data are means ± SEM. *p < 0.05, **p < 0.01, ***p < 0.001 between indicated datasets

We next investigated how changes in [Cl^−^]_o_ were transduced into a change in CFTR activity. Because gating of phosphorylated CFTR is regulated by rounds of ATP binding and hydrolysis at the two NBDs [36, 16, 23], we first focussed on the role of ATP hydrolysis. Non-hydrolysable ATP analogues are known to ‘lock’ PKA phosphorylated CFTR into the open state upon binding to ATP binding site 2 on NBD2 (the site where rapid ATP hydrolysis normally occurs) [36, 16]. We first tested the effect of adding 1 mM adenosine 5′-(β,γ-imido)triphosphate lithium salt hydrate (AMP-PNP) to the intracellular (pipette) solution, in the presence of 1 mM ATP, with total MgCl_2_ increased to 3 mM, on WT channel responses. Note that increasing the pipette MgCl_2_ to 3 mM on its own had no significant effect on the degree of stimulation by [Cl^−^]_o_ (high Cl^−^ stimulation; 56.7 ± 8.9 %, *n* = 7). We confirmed that AMP-PNP was capable of locking open CFTR under these conditions because the rate of current decay following removal of FSK was markedly extended in the presence of AMP-PNP (Fig. 3a). Figure 3b, c and f show that AMP-PNP eliminated the [Cl^−^]_o_-dependent activation of FSK-stimulated WT CFTR (high Cl^−^ stimulation; −1.7 ± 3.7 %, *n* = 5).

We then examined whether the ATP hydrolysis-deficient CFTR mutant, E1371Q [38], could sense [Cl^−^]_o_ with the expectation that it would not. Like WT CFTR exposed to AMP-PNP, the E1371Q mutant channels display extended open channel bursts in the presence of ATP [16, 23, 40]. In our experiments, E1371Q CFTR expressed in HEK cells was constitutively active and large currents were detected without prior stimulation with FSK (Fig. 3d (i) (first set of fWCR current traces), and 3e), which agrees with previous work performed in baby hamster kidney (BHK) cells [27, 42, 46]. The reason for this constitutive activity of E1371Q is unclear and it contrasts with the behaviour of wild type CFTR and other mutants (except DeltaR-CFTR) previously investigated in our laboratories using the BHK and HEK cell expression systems [46]. Exposing E1371Q CFTR (without FSK) to an increase in [Cl^−^]_o_ caused only a small stimulation of channel activity (21.9 ± 14.1 %, *n* = 6; Fig. 3f). Surprisingly in view of the WT CFTR data with AMP-PNP, if E1371Q CFTR channels were first phosphorylated with FSK (which itself had no significant effect on the size of the whole cell currents (Fig. 3d (ii) (second set of fWCR traces)) and Table 1), [Cl^−^]_o_ sensing was restored (174.5 ± 56.2 % stimulation, *n* = 8; Fig. 3d, e and f).

To confirm the role of phosphorylation in [Cl^−^]_o_ sensing, we reduced the endogenous phosphorylation of E1371Q channels by removing ATP/GTP from the intracellular solution *prior* to stimulation with FSK, which significantly reduced basal E1371Q CFTR currents (No ATP/GTP: −186 ± 122 pA/pF *n* = 8 vs ATP/GTP: −1,259 ± 433 pA/pF, *n* = 8, *P* < 0.001). Under these conditions, the E1371Q CFTR channels failed to respond to changes in [Cl^−^]_o_ even in the presence of FSK (Fig. 3f), consistent with the idea that only phosphorylated channels are gated by [Cl^−^]_o_. Since E1371Q CFTR is hydrolysis-deficient [38], we expected that [Cl^−^]_o_ sensing by the mutant would not be affected by AMP-PNP. However, unexpectedly, we found that the ability of FSK-stimulated E1371Q CFTR channels to respond to changes in [Cl^−^]_o_ was markedly reduced by the non-hydrolysable ATP analogue, just like WT CFTR (Fig. 3f).

To check that the restoration of [Cl^−^]_o_ sensing by E1371Q CFTR after phosphorylation (Fig. 3d) was due to an interaction of Cl^−^ with the extracellular domain of CFTR, we also studied the double-mutant R899Q-E1371Q. Figure 3f shows that [Cl^−^]_o_ sensing by the double mutant in the presence of FSK was significantly reduced compared to E1371Q CFTR under the same conditions, confirming that phosphorylated E1371Q CFTR channels respond to changes in [Cl^−^]_o_ via R899 in ECL4.

**Fig. 4:**
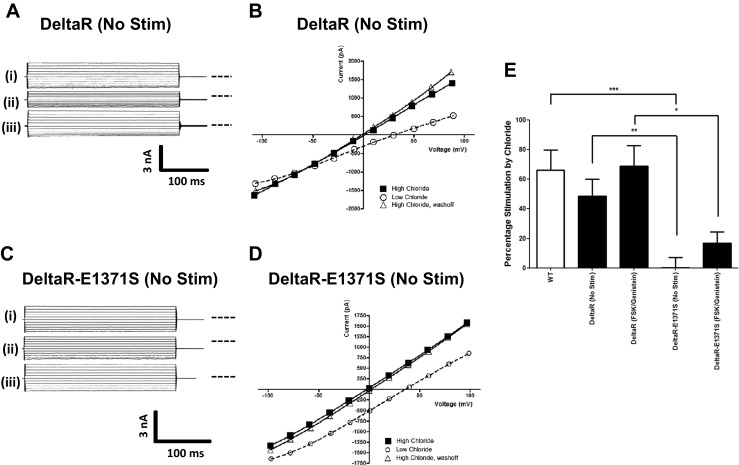
Role of the R domain in [Cl^−^]_o_ sensing by CFTR. **a, c** Representative fWCR current recordings measured between ±100 mV in 20 mV steps from HEK cells transfected with deltaR-CFTR or deltaR-E1371S CFTR as indicated. Note all experiments used unstimulated conditions. The current traces are from the top down: (*i*) unstimulated in 155.5 mM [Cl^−^]_o_, (*ii*) unstimulated in 35.5 mM [Cl^−^]_o_ and (*iii*) unstimulated in 155.5 mM [Cl^−^]_o_. *Dotted line* to the right of the current traces indicates zero current level. **b, d** Representative I-V plots for the data presented in **a** and **c. e** Percentage current stimulation by [Cl^−^]_o_ for WT CFTR (*n* = 24) and for DeltaR and DeltaR-E1371Q mutants (see Fig. 1) under unstimulated (*No Stim*) or after exposure to a combination of forskolin and genistein (FSK/Genistein). (*n* = 4–10). Data are means ± SEM. *p < 0.05, **p < 0.01 compared to indicated datasets

To explore the role of phosphorylation further, we studied the effect of deleting the R domain from CFTR (residues 634–836) [12, 7], which removes all the major PKA/PKC consensus phosphorylation sites [36, 32]. Unlike WT CFTR, DeltaR-CFTR channels are spontaneously active without PKA phosphorylation, but are gated normally by cytosolic ATP [12, 7]. Note that because this mutant does not express very well in HEK cells, we also included 30 μM genistein (a CFTR potentiator, [1]) with FSK to increase the activity of DeltaR-CFTR channels. Genistein itself had no effect on the ability of DeltaR-CFTR (nor WT CFTR) to respond to changes in [Cl^−^]_o_ (data not shown), so the FSK and genistein results have been combined. Figure 4a–e shows that both in the absence and presence of stimulants (FSK + Genistein), DeltaR-CFTR exhibited WT-like levels of [Cl^−^]_o_ stimulation, indicating that deleting the R domain removes the phosphorylation-dependence of [Cl^−^]_o_ sensing observed for WT CFTR (Fig. 2). Based on our previous result that the phosphorylated, hydrolysis-deficient, E1371Q mutant could sense [Cl^−^]_o_ (Fig. 3f), we predicted that preventing ATP hydrolysis at ATP binding site 2 of DeltaR-CFTR would have no effect on [Cl^−^]_o_ sensing. We tested this prediction by using DeltaR-CFTR with the E1371S mutation, which like E1371Q CFTR is hydrolysis defective [8] and found, unexpectedly, that the double-mutant CFTR channels did not respond to changes in [Cl^−^]_o_ (external Cl^−^ stimulation; no FSK/genistein: 0.0 ± 7.0 %, *n* = 9; with FSK/genistein: 16.7 ± 7.7 %, *n* = 4) (Fig. 4c–e).

### Role of ATP binding and ATPase activity of the NBDs in [Cl^−^]_o_ sensing by CFTR

**Fig. 5:**
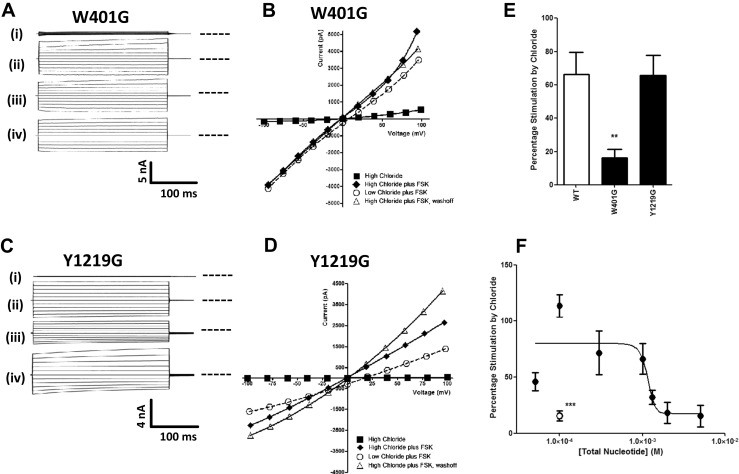
ATP binding to site 1, but not site 2, underlies [Cl^−^]_o_ sensing by CFTR. **a**, **c** Representative fWCR current recordings measured between ±100 mV in 20 mV steps from HEK cells transfected with W401G CFTR or Y1219G CFTR, as indicated. The current traces are from the top down: (*i*) unstimulated in 155.5 mM [Cl^−^]_o_, (*ii*) forskolin (*FSK*)-stimulated in 155.5 mM [Cl^−^]_o_, (*iii*) FSK-stimulated in 35.5 mM [Cl^−^]_o_ and (*iv*) FSK-stimulated in 155.5 mM [Cl^−^]_o_. *Dotted line* to the right of the current traces indicates zero current level. **b**, **d** Representative I-V plots for the data presented in **a** and **c. e** Percentage current stimulation by [Cl^−^]_o_ for WT CFTR (*n* = 24) and for W401G (NBD1) and Y1219G (NBD2) mutants (see Fig. 1) (*n* = 7–8). Data are mean ± SEM. **p < 0.01 compared to WT CFTR. **f** Percentage current stimulation by [Cl^−^]_o_ for WT CFTR at different cytosolic (pipette) ATP concentrations. Data are means ± SEM (*n* = 5–24). Data points fitted using least squares fit of a log [inhibition] vs response equation, with variable slope function and with no constraints or weighting. The *open circle* shows the effect of including 50 μM P-ATP with 50 μM ATP on the response to [Cl^−^]_o_. ***p < 0.001 compared to 100 μM ATP

Figures 3 and 4 suggest that the role of ATP hydrolysis in [Cl^−^]_o_ sensing by CFTR is complex; e.g. AMP-PNP abolishes [Cl^−^]_o_ sensing by WT CFTR, whereas the E1371Q mutation has no effect. Possibly changes in ATP interaction with the NBDs, and/or subsequent NBD dimerisation, rather than just hydrolysis, could be primarily responsible for the effects of [Cl^−^]_o_. To test this idea, we individually mutated key residues involved in ATP binding at site 1 in NBD1 (W401) and site 2 in NBD2 (Y1219) to glycine (Fig. 1), and then measured responses to changes in [Cl^−^]_o_. Note that both of these mutations substantially reduce CFTR channel activity through mechanisms that are not secondary to changes in ATP hydrolysis rate [49], and in the case of W401G, not due to an apparent change in ATP binding affinity. Sub-panels a, b and e of Fig. 5 show that the FSK-stimulated W401G CFTR mutant exhibited significantly reduced [Cl^−^]_o_ sensing compared to WT CFTR, whereas the FSK-stimulated Y1219G CFTR mutant did not (Fig. 5c–e).

We then reasoned that if events downstream of ATP binding to site 1, and/or NBD dimerisation, were altered by changes in [Cl^−^]_o_ (as the W401G data suggested), then the effect of [Cl^−^]_o_ on CFTR activity should be sensitive to the concentration of cytosolic ATP. Figure 5f shows that this was indeed the case. [Cl^−^]_o_ sensing by CFTR was gradually abolished as cytosolic [ATP] was increased to ∼2 mM, with an estimated IC_50_ of 1.15 ± 0.14 mM, and a maximal stimulation of 80.0 ± 11.3 %. Consistent with the idea that ATP binding energy and/or NBD dimerisation was being altered by [Cl^−^]_o_, we also investigated the effect of including a higher affinity ATP analogue, N^6^-(2-phenylethyl)-ATP (P-ATP, [48]), with a low dose of ATP (50 μM). The presence of P-ATP (50 μM) significantly reduced the ability of WT CFTR to respond to changes in [Cl^−^]_o_ when compared to the equivalent control (100 μM ATP) response (Fig. 5f, open circle), further implicating ATP/NBD interaction as a key factor in the activation of CFTR by high [Cl^−^]_o_.

**Fig. 6:**
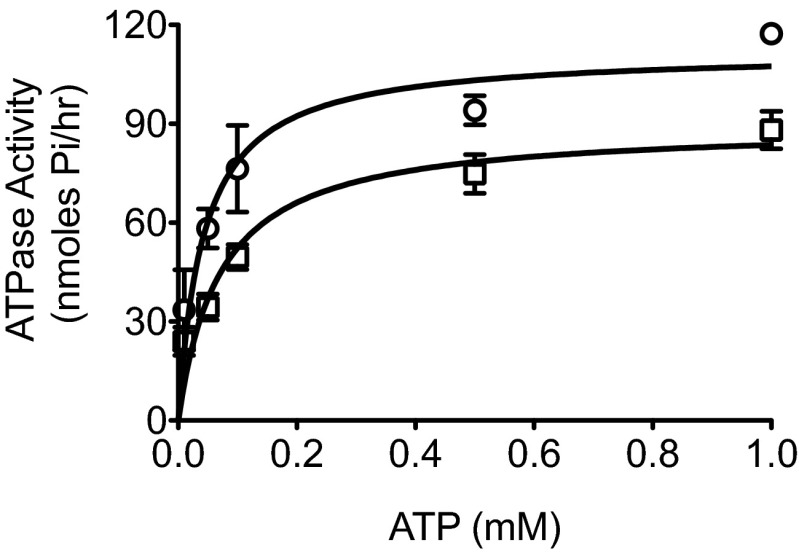
ATPase activity of CFTR is stimulated by a decrease in Cl^−^ concentration. Purified WT CFTR was reconstituted into dodecylmaltoside (DDM) micelles in the presence of either 150 mM (*squares*) or 50 mM Cl^−^ (*circles*) and the ATPase activity measured over a range of ATP concentrations, as described in the “Materials and methods.” Data are means ± SEM for 3 (low Cl^−^) or 4 (high Cl^−^) separate purification/reconstitution experiments. Data points fitted using the Michaelis Menten equation (*r* ^2^ = 0.83 (NaCl) and *r* ^2^ = 0.8 (NaGlu))

If high [Cl^−^]_o_ activates CFTR by stabilising the NBD1-NBD2 dimer, as suggested by the results in Fig. 5, we reasoned that the ATPase activity of WT CFTR (which is mostly conferred by site 2; [38, 6]) should be reduced by high [Cl^−^]. To test this prediction, we performed ATPase measurements on purified CFTR reconstituted into micelles and pre-phosphorylated by PKA as previously described [15], over a range of [ATP]. Figure 6 shows that when total [Cl^−^] was increased from 50 to 150 mM (with total salt kept constant at 150 mM using Na-gluconate as a replacement for NaCl), there was a marked shift in the ATPase activity curve, such that at over the range of [ATP] up to 1.0 mM, there was a significant decrease in ATPase activity. This change in ATPase activity was due to a reduction in ATP turnover (*V*_max_ for low [Cl^−^]; 114.3 ± 1.5 nmol/h, *n* = 3, versus high [Cl^−^]; 89.3 ± 7.3 nmol/h, *n* = 4, *p* < 0.04), without a significant change in apparent binding affinity (*K*_m_) of CFTR for ATP (*K*_m_ for low [Cl^−^]; 48 ± 12 μM, *n* = 3 versus high [Cl^−^]; 69 ± 16 μM, *n* = 4, *p* > 0.05).

## Discussion

Our data provide the first evidence that [Cl^−^]_o_-dependent stimulation of CFTR is an intrinsic property of the channel, and that R899 located in ECL4 is absolutely essential for the response. Although neutralization of the positive charge at R899, as well as charge reversal, eliminated [Cl^−^]_o_ sensing by CFTR (Fig. 2), we have no direct evidence that Cl^−^ ions actually bind to this residue. Nevertheless, we assume that R899 must reside somewhere in the pathway between Cl^−^ binding and enhanced CFTR activity. In support of this conclusion, the stimulating effect of [Cl^−^]_o_ cannot be due to the ion entering the pore, since mutations that do affect CFTR Cl^−^ conductance (R104Q, R117Q, R334Q, K335A) have no effect on [Cl^−^]_o_ stimulation (Fig. 2), and the one site that is important for sensing (R899) does not affect Cl^−^ conductance [45, 47]. It is conceivable that the R899Q/E mutations cause abnormal folding of CFTR, which results in a change in the interaction between Cl^−^ and some other residue in the protein. However, we noticed no major differences in the expression levels of WT CFTR and the R899 mutants, nor any effects of R899 mutations on CFTR channel properties; e.g. cAMP activation, which would be expected if there was a major folding problem [45, 47].

We can make a number of conclusions about the molecular mechanism involved in the [Cl^−^]_o_ stimulation of CFTR. First, cAMP/PKA-dependent phosphorylation is required for the response in WT and E1371Q CFTR channels. However, since [Cl^−^]_o_ sensing still occurred when the R domain was deleted (Fig. 4), the ability to respond to [Cl^−^]_o_ must reside in other domains. In intact CFTR, these are likely to be parts of the protein that are regulated by the R domain once it is phosphorylated. Although the R domain is a relatively disordered structure, there is evidence from NMR studies that regions of the R domain interact with the NBDs [4, 9]. Upon phosphorylation of the R domain, it is thought that these regions of interaction change and that this allows the NBDs to dimerise in the presence of ATP, thereby promoting channel opening [36, 16, 23, 4, 9]. Therefore, in terms of facilitating [Cl^−^]_o_ sensing, both phosphorylation of the R domain and its deletion are functionally equivalent (but see below).

Secondly, in phosphorylated CFTR channels, impairing ATP hydrolysis using the E1371Q mutation did not prevent [Cl^−^]_o_ sensing. This result implies that chloride sensing does not lead to a change in channel gating simply via a change in ATPase activity. Although chloride sensing does cause a modest change in intrinsic ATPase activity (Fig. 6), this cannot account for the larger effects on channel gating. The fact that [Cl^−^]_o_ sensing is impaired in phosphorylated E1371Q CFTR channels when AMP-PNP is present, is probably explained by AMP-PNP substituting for ATP in its actions. As Fig. 5f shows, raising cytosolic [ATP] to 2 mM, or above, abolished sensing. This is why AMP-PNP effects are mimicked by ATP, but not by the E1371Q mutation. In this context, it is important to remember that although the E1371Q mutation substantially reduces ATP hydrolysis (∼70 %, [38]), the channel is still gated by ATP, even in a DeltaR background [12, 7, 8]. The one caveat to this interpretation is that [Cl^−^]_o_ sensing by DeltaR-CFTR was abolished when ATP hydrolysis at NBD2 was blocked by the E1371S mutation. This result indicates that intra-domain interactions in CFTR that lacks an R domain cannot fully recapitulate those in WT CFTR, despite evidence that DeltaR-CFTR has similar gating properties to WT CFTR [12, 7].

Thirdly, the fact that [Cl^−^]_o_ sensing is abolished by the W401G mutation in NBD1, but not by the corresponding NBD2 mutant, Y1219G (Fig. 5e), suggests that ATP binding at NBD1, and not NBD2, is mainly responsible for transducing the [Cl^−^]_o_-dependent gating changes. Furthermore, since P-ATP also prevented [Cl^−^]_o_ from changing CFTR activity (Fig. 5f), then our results are best explained by external Cl^−^ affecting the stability of the NBD1/NBD2 dimer (more stable at higher external Cl^−^). Thus both the presence of cytosolic P-ATP as well high [Cl^−^]_o_ converge on the same process.

Fourthly, changes in Cl^−^ concentration, at constant Na^+^, alters the intrinsic ATPase activity of intact CFTR by ∼20 %, which argues for a direct effect of Cl^−^ on the intrinsic catalytic activity of the protein (Fig. 6). This result shows that in addition to total salt concentration [24], Cl^−^ concentration alone can regulate the ATPase activity of purified CFTR and appears to do this by altering the ATP turnover (hydrolysis) rate by the NBDs. The mechanism for this change in ATP turnover is uncertain at the present time. In addition, whether substitution of arginine for glutamine at position 899 in ECL4 leads to a change in ATPase activity of the CFTR is an important area for future research.

In conclusion, our results support a mechanism whereby an increase in [Cl^−^]_o_ stimulates CFTR activity by modulating ATP binding/interaction at NBD1, which favours NBD dimerisation, reducing ATP hydrolysis and hence increasing channel activity. Thus our results suggest that the increased P_o_ of the CFTR channel that we reported previously under high [Cl^−^]_o_ conditions [44] is due to external Cl^−^ acting at ECL4 to alter single channel gating. In addition, and in contrast to the known effects on P_o_, an effect of external Cl^−^ on the number of active channels (N) and the single channel current (i) can effectively be ruled out. Thus the current paradigm that transmission of ‘gating information’ in phosphorylated CFTR is temporally and spatially directed from the cytosolic NBDs to the TMDs needs to be revised. Specifically, our data shows that an interaction of Cl^−^ with externally accessible parts of the TMDs also feeds back to modulate NBD function. How R899 in ECL4 alters CFTR gating is unclear; however, ECL4 connects transmembrane segments 7 and 8 in TMD2 and is the largest ECL in CFTR and exhibits considerable flexibility (Fig. 1b). Recent molecular modelling and cross-linking experiments [21, 28, 34] predict that there are inter-domain interactions between the TMDs and their opposite NBDs through the intracellular loops (ICLs), which act as coupling helices. Since TM segment 8 is connected to TM segment 9 via ICL3, and ICL3 has been shown to lie in close proximity to NBD1, and to interact with the NBD-ATP binding sites [28], then it is conceivable that Cl^−^-dependent alterations in ECL4 are transmitted to NBD1 via ICL3.

Altogether, our present results, together with previous studies [30, 20], demonstrate that the short ECLs of CFTR are important for controlling channel activity. However, this study is the first to provide an explanation for how an ECL residue can alter CFTR gating via changes in the interaction of cytosolic ATP with CFTR NBDs. Interestingly, a recent study by Cui et al. [13] has shown that three charged amino acid residues in ECL1 (D110, E116 and R117) are involved in stabilising the architecture of the outer pore of CFTR by interacting with other charged residues, including K892 in ECL4. Furthermore, our lab has recently demonstrated, through cross-linking of residues in ECL1 and ECL4, that the relative movement of these two ECLs is necessary for normal channel gating [10] which further demonstrates the important role of the ECLs in CFTR channel function.

We speculate that [Cl^−^]_o_ sensing may be a feedback mechanism allowing CFTR to orchestrate anion and fluid secretion in epithelial tissues that secrete an isotonic mixture of Cl^−^ and HCO_3_^−^ [25, 33, 37, 39, 43]. For instance, in pancreatic ducts, luminal [Cl^−^] falls as luminal [HCO_3_^−^] rises which would limit CFTR activity and favour hyperpolarization of the luminal membrane, thus maintaining an electrical driving force for HCO_3_^−^ secretion via either electrogenic SLC26 family anion exchangers [26] or CFTR itself [3]. However, in tissues that are thought to secrete little HCO_3_^−^, such as the surface cells lining the larger airways and nasal tract [17, 11], where ASL [Cl^−^] is likely to remain high, then the mechanism we describe is less likely to be of major significance. Finally, the [Cl^−^]_o_ sensing site in CFTR that includes R899 on its extracellular face is a potential drug target for the correction of CF-causing gating mutants if a compound could be developed that mimicked the stimulatory effect of [Cl^−^]_o_ on the channel.

## References

[1] Al-Nakkash L, Hu S, Li M, Hwang TC (2001). A common mechanism for cystic fibrosis transmembrane conductance regulator protein activation by genistein and benzimidazolone analogs. J Pharmacol Exp Ther.

[2] Aleksandrov L, Aleksandrov AA, Chang XB, Riordan JR (2002). The first nucleotide binding domain of cystic fibrosis transmembrane conductance regulator is a site of stable nucleotide interaction, whereas the second is a site of rapid turnover. J Biol Chem.

[3] Argent BE, Gray MA, Steward MC, Case RM (2012) In: L Johnson (ed) Physiology of the Gastrointestinal Tract. 5th edn. Elsevier, San Diego, pp 1399–1423

[4] Baker JM, Hudson RP, Kanelis V, Choy WY, Thibodeau PH, Thomas PJ, Forman-Kay JD (2007). CFTR regulatory region interacts with NBD1 predominantly via multiple transient helices. Nat Struct Mol Biol.

[5] Basso C, Vergani P, Nairn AC, Gadsby DC (2003). Prolonged nonhydrolytic interaction of nucleotide with CFTR’s NH2-terminal nucleotide binding domain and its role in channel gating. J Gen Physiol.

[6] Berger AL, Ikuma M, Welsh MJ (2005). Normal gating of CFTR requires ATP binding to both nucleotide-binding domains and hydrolysis at the second nucleotide-binding domain. Proc Natl Acad Sci U S A.

[7] Bompadre SG, Ai T, Cho JH, Wang X, Sohma Y, Li M, Hwang TC (2005). CFTR gating I: characterization of the ATP-dependent gating of a phosphorylation-independent CFTR channel (DeltaR-CFTR). J Gen Physiol.

[8] Bompadre SG, Cho JH, Wang X, Zou X, Sohma Y, Li M, Hwang TC (2005). CFTR gating II: effects of nucleotide binding on the stability of open states. J Gen Physiol.

[9] Bozoky Z, Krzeminski M, Muhandiram R, Birtley JR, Al-Zahrani A, Thomas PJ, Frizzell RA, Ford RC, Forman-Kay JD (2013). Regulatory R region of the CFTR chloride channel is a dynamic integrator of phospho-dependent intra- and intermolecular interactions. Proc Natl Acad Sci U S A.

[10] Broadbent SD, Wang W, Linsdell P (2014). Interaction between two extracellular loops influences the activity of the cystic fibrosis transmembrane conductance regulator chloride channel. Biochem Cell Biol.

[11] Cho DY, Hwang PH, Illek B, Fischer H (2011). Acid and base secretion in freshly excised nasal tissue from cystic fibrosis patients with DeltaF508 mutation. Int Forum Allergy Rhinol.

[12] Csanady L, Chan KW, Seto-Young D, Kopsco DC, Nairn AC, Gadsby DC (2000). Severed channels probe regulation of gating of cystic fibrosis transmembrane conductance regulator by its cytoplasmic domains. J Gen Physiol.

[13] Cui G, Rahman KS, Infield DT, Kuang C, Prince CZ, McCarty NA (2014). Three charged amino acids in extracellular loop 1 are involved in maintaining the outer pore architecture of CFTR. J Gen Physiol.

[14] Dalton J, Kalid O, Schushan M, Ben-Tal N, Villa-Freixa J (2012). New model of cystic fibrosis transmembrane conductance regulator proposes active channel-like conformation. J Chem Inf Model.

[15] Eckford PD, Li C, Ramjeesingh M, Bear CE (2012). Cystic fibrosis transmembrane conductance regulator (CFTR) potentiator VX-770 (ivacaftor) opens the defective channel gate of mutant CFTR in a phosphorylation-dependent but ATP-independent manner. J Biol Chem.

[16] Gadsby DC, Vergani P, Csanady L (2006). The ABC protein turned chloride channel whose failure causes cystic fibrosis. Nature.

[17] Garland AL, Walton WG, Coakley RD, Tan CD, Gilmore RC, Hobbs CA, Tripathy A, Clunes LA, Bencharit S, Stutts MJ, Betts L, Redinbo MR, Tarran R (2013). Molecular basis for pH-dependent mucosal dehydration in cystic fibrosis airways. Proc Natl Acad Sci U S A.

[18] Gong X, Burbridge SM, Cowley EA, Linsdell P (2002). Molecular determinants of Au(CN)(2)(−) binding and permeability within the cystic fibrosis transmembrane conductance regulator Cl(−) channel pore. J Physiol.

[19] Gong X, Linsdell P (2003). Molecular determinants and role of an anion binding site in the external mouth of the CFTR chloride channel pore. J Physiol.

[20] Hammerle MM, Aleksandrov AA, Riordan JR (2001). Disease-associated mutations in the extracytoplasmic loops of cystic fibrosis transmembrane conductance regulator do not impede biosynthetic processing but impair chloride channel stability. J Biol Chem.

[21] He L, Aleksandrov AA, Serohijos AW, Hegedus T, Aleksandrov LA, Cui L, Dokholyan NV, Riordan JR (2008). Multiple membrane-cytoplasmic domain contacts in the cystic fibrosis transmembrane conductance regulator (CFTR) mediate regulation of channel gating. J Biol Chem.

[22] Heda GD, Marino CR (2000). Surface expression of the cystic fibrosis transmembrane conductance regulator mutant DeltaF508 is markedly upregulated by combination treatment with sodium butyrate and low temperature. Biochem Biophys Res Commun.

[23] Jih KY, Hwang TC (2012). Nonequilibrium gating of CFTR on an equilibrium theme. Physiology Bethesda.

[24] Kogan I, Ramjeesingh M, Huan LJ, Wang Y, Bear CE (2001). Perturbation of the pore of the cystic fibrosis transmembrane conductance regulator (CFTR) inhibits its ATPase activity. J Biol Chem.

[25] Kopelman H, Corey M, Gaskin K, Durie P, Weizman Z, Forstner G (1988). Impaired chloride secretion, as well as bicarbonate secretion, underlies the fluid secretory defect in the cystic fibrosis pancreas. Gastroenterology.

[26] Lee MG, Ohana E, Park HW, Yang D, Muallem S (2012). Molecular mechanism of pancreatic and salivary gland fluid and HCO3 secretion. Physiol Rev.

[27] Li MS, Holstead RG, Wang W, Linsdell P (2011). Regulation of CFTR chloride channel macroscopic conductance by extracellular bicarbonate. Am J Physiol.

[28] Mornon JP, Lehn P, Callebaut I (2008). Atomic model of human cystic fibrosis transmembrane conductance regulator: membrane-spanning domains and coupling interfaces. Cell Mol Life Sci.

[29] O’Reilly CM, Winpenny JP, Argent BE, Gray MA (2000). Cystic fibrosis transmembrane conductance regulator currents in guinea pig pancreatic duct cells: inhibition by bicarbonate ions. Gastroenterology.

[30] Price MP, Ishihara H, Sheppard DN, Welsh MJ (1996). Function of Xenopus cystic fibrosis transmembrane conductance regulator (CFTR) Cl channels and use of human-Xenopus chimeras to investigate the pore properties of CFTR. J Biol Chem.

[31] Ramjeesingh M, Ugwu F, Stratford FL, Huan LJ, Li C, Bear CE (2008). The intact CFTR protein mediates ATPase rather than adenylate kinase activity. Biochem J.

[32] Riordan JR, Rommens JM, Kerem B, Alon N, Rozmahel R, Grzelczak Z, Zielenski J, Lok S, Plavsic N, Chou JL (1989). Identification of the cystic fibrosis gene: cloning and characterization of complementary DNA. Science (New York, NY).

[33] Robinson PJ, Smith AL, Sly PD (1990). Duodenal pH in cystic fibrosis and its relationship to fat malabsorption. Dig Dis Sci.

[34] Serohijos AW, Hegedus T, Aleksandrov AA, He L, Cui L, Dokholyan NV, Riordan JR (2008). Phenylalanine-508 mediates a cytoplasmic-membrane domain contact in the CFTR 3D structure crucial to assembly and channel function. Proc Natl Acad Sci U S A.

[35] Shcheynikov N, Kim KH, Kim KM, Dorwart MR, Ko SB, Goto H, Naruse S, Thomas PJ, Muallem S (2004). Dynamic control of cystic fibrosis transmembrane conductance regulator Cl(−)/HCO3(−) selectivity by external Cl(−). J Biol Chem.

[36] Sheppard DN, Welsh MJ (1999). Structure and function of the CFTR chloride channel. Physiol Rev.

[37] Smith JJ, Welsh MJ (1992). cAMP stimulates bicarbonate secretion across normal, but not cystic fibrosis airway epithelia. J Clin Invest.

[38] Stratford FL, Ramjeesingh M, Cheung JC, Huan LJ, Bear CE (2007). The Walker B motif of the second nucleotide-binding domain (NBD2) of CFTR plays a key role in ATPase activity by the NBD1-NBD2 heterodimer. Biochem J.

[39] Thiagarajah JR, Song Y, Haggie PM, Verkman AS (2004). A small molecule CFTR inhibitor produces cystic fibrosis-like submucosal gland fluid secretions in normal airways. Faseb J.

[40] Vergani P, Lockless SW, Nairn AC, Gadsby DC (2005). CFTR channel opening by ATP-driven tight dimerization of its nucleotide-binding domains. Nature.

[41] Verkman AS, Galietta LJ (2009) Chloride channels as drug targets. Nat Rev Drug Discov 8(2):153–171 10.1038/nrd2780 PMC3601949 19153558

[42] Wang W, Linsdell P (2012). Alternating access to the transmembrane domain of the ATP-binding cassette protein cystic fibrosis transmembrane conductance regulator (ABCC7). J Biol Chem.

[43] Wang XF, Zhou CX, Shi QX, Yuan YY, Yu MK, Ajonuma LC, Ho LS, Lo PS, Tsang LL, Liu Y, Lam SY, Chan LN, Zhao WC, Chung YW, Chan HC (2003). Involvement of CFTR in uterine bicarbonate secretion and the fertilizing capacity of sperm. Nat Cell Biol.

[44] Wright AM, Gong X, Verdon B, Linsdell P, Mehta A, Riordan JR, Argent BE, Gray MA (2004). Novel regulation of cystic fibrosis transmembrane conductance regulator (CFTR) channel gating by external chloride. J Biol Chem.

[45] Zhou JJ, Fatehi M, Linsdell P (2008). Identification of positive charges situated at the outer mouth of the CFTR chloride channel pore. Pflugers Arch.

[46] Zhou JJ, Li MS, Qi J, Linsdell P (2010). Regulation of conductance by the number of fixed positive charges in the intracellular vestibule of the CFTR chloride channel pore. J Gen Physiol.

[47] Zhou JJ, Linsdell P (2009). Evidence that extracellular anions interact with a site outside the CFTR chloride channel pore to modify channel properties. Can J Physiol Pharmacol.

[48] Zhou Z, Wang X, Li M, Sohma Y, Zou X, Hwang TC (2005). High affinity ATP/ADP analogues as new tools for studying CFTR gating. J Physiol.

[49] Zhou Z, Wang X, Liu HY, Zou X, Li M, Hwang TC (2006). The two ATP binding sites of cystic fibrosis transmembrane conductance regulator (CFTR) play distinct roles in gating kinetics and energetics. J Gen Physiol.

